# Glutamatergic deficits and parvalbumin-containing inhibitory neurons in the prefrontal cortex in schizophrenia

**DOI:** 10.1186/1471-244X-9-71

**Published:** 2009-11-16

**Authors:** BKY Bitanihirwe, MP Lim, JF Kelley, T Kaneko, TUW Woo

**Affiliations:** 1 Laboratory of Cellular Neuropathology, McLean Hospital, Belmont, MA, USA; 2 Department of Psychiatry, Harvard Medical School, Boston, MA, USA; 3 Department of Psychiatry, Beth Israel Deaconess Medical Center, Boston, MA, USA; 4 Department of Morphological Brain Science, Kyoto University, Kyoto, Japan; 5 Laboratory of Behavioral Neurobiology, ETH Zurich, Schorenstrasse 16, Schwerzenbach 8603, Switzerland

## Abstract

**Background:**

We have previously reported that the expression of the messenger ribonucleic acid (mRNA) for the NR2A subunit of the N-methyl-D-aspartate (NMDA) class of glutamate receptor was decreased in a subset of inhibitory interneurons in the cerebral cortex in schizophrenia. In this study, we sought to determine whether a deficit in the expression of NR2A mRNA was present in the subset of interneurons that contain the calcium buffer parvalbumin (PV) and whether this deficit was associated with a reduction in glutamatergic inputs in the prefrontal cortex (PFC) in schizophrenia.

**Methods:**

We examined the expression of NR2A mRNA, labeled with a ^35^S-tagged riboprobe, in neurons that expressed PV mRNA, visualized with a digoxigenin-labeled riboprobe via an immunoperoxidase reaction, in twenty schizophrenia and twenty matched normal control subjects. We also immunohistochemically labeled the glutamatergic axon terminals with an antibody against vGluT1.

**Results:**

The density of the PV neurons that expressed NR2A mRNA was significantly decreased by 48-50% in layers 3 and 4 in the subjects with schizophrenia, but the cellular expression of NR2A mRNA in the PV neurons that exhibited a detectable level of this transcript was unchanged. In addition, the density of vGluT1-immunoreactive boutons was significantly decreased by 79% in layer 3, but was unchanged in layer 5 of the PFC in schizophrenia.

**Conclusion:**

These findings suggest that glutamatergic neurotransmission via NR2A-containing NMDA receptors on PV neurons in the PFC may be deficient in schizophrenia. This may disinhibit the postsynaptic excitatory circuits, contributing to neuronal injury, aberrant information flow and PFC functional deficits in schizophrenia.

## Background

The prefrontal cortex (PFC) plays an important role in the temporal organization of behavior [1,2] by temporarily maintaining "on-line" internal representations of perceptual, cognitive and emotive information in order to guide sequential, contextually meaningful behavior [3]. This "executive functioning" capacity forms the basis of many daily human activities, such as planning, reasoning and thinking, and is known to be impaired in patients with schizophrenia [4].

Sustained discharge of neuronal circuits is thought to be the physiological substrate that mediates on-line maintenance and manipulation of information performed by the PFC [1,2]. Inhibitory neurons that utilize γ-aminobutyric acid (GABA) as neurotransmitter play a key role in regulating sustained neuronal activation by dynamically adjusting the conductances of the pyramidal neuronal network. Importantly, the number of N-methyl-D-aspartate (NMDA) glutamate receptors within a neural network, including those that are localized on GABA neurons, appears to be a critical determinant of the stability of the network in sustaining neuronal activation [5-9]. GABA neurons receive feedback excitatory modulation via local recurrent excitatory projections from the pyramidal neurons they innervate and, at the same time, they are also targets of feedforward excitatory modulation from axonal projections furnished by other pyramidal neurons, located both within the PFC and in other cortical areas [10-12]. The integrity of PFC functions therefore depends on the delicate interplay of feedback and feedforward mechanisms of modulation of cortical inhibitory activities via activation of glutamate receptors on GABA interneurons [8,9,13,14].

There has been compelling evidence suggesting that GABA neurons in the PFC are functionally disturbed in schizophrenia [15-18]. In addition, disturbances of glutamatergic modulation of these neurons could further compromise their normal functioning. In fact, we have recently found that, in both the PFC and anterior cingulate cortex, the expression of the mRNA for the NR2A subunit of the NMDA glutamate receptor was decreased to a level that was no longer experimentally detectable in 49-73% of the GABA neurons that normally expressed this transcript in subjects with schizophrenia [19,20]. Because connectionally and functionally distinct subpopulations of GABA neurons regulate different aspects of information flow in the cerebral cortex [21-23], an important question that must be addressed in order to truly appreciate the pathophysiologic consequences of altered glutamatergic modulation of GABA neuronal functions in schizophrenia is the identity of the GABA cells that are affected.

Increasing evidence suggests that the subset of GABA cells that contain the calcium buffering protein parvalbumin (PV), which exhibit fast-spiking firing properties and target the perisomatic (basket cells) and axo-axonic (chandelier cells) compartments of pyramidal neurons [24,25], are functionally disturbed in schizophrenia [17,26], and these cells express NR2A [27-29]. In this study, using double *in situ *hybridization, we found that the density of NR2A mRNA-expressing PV neurons was decreased by as much as 50% in subjects with schizophrenia in a layer-specific manner. In addition, we immunohistochemically labeled glutamatergic terminals with an antibody against the vesicular glutamate transporter vGluT1 [30,31]. We found that the density of these terminals also exhibited a reduction with a laminar pattern that paralleled the reduction in the NR2A-expressing PV neurons. Together these observations suggest that glutamatergic innervation of PV-containing inhibitory neurons appears to be deficient in schizophrenia.

## Methods

### Human Subjects

Post-mortem brains from subjects whose next of kin had given consent for their tissues to be used in medical research were obtained from the Harvard Brain Tissue Resource Center at McLean Hospital, Belmont, Massachusetts. The informed consent process has been approved by the McLean Hospital Human Research Committee. Comparison group brains were collected from subjects diagnosed with schizophrenia (*n *= 20) and normal control subjects (*n *= 20) matched for age, postmortem interval (PMI), brain pH and wherever possible, sex and hemispheric laterality. All brains were examined by a neuropathologist to rule out any neurologic conditions (Additional file 1).

Diagnosis of schizophrenia was made by reviewing medical records and an extensive family questionnaire that included medical, psychiatric and social history. Two psychiatrists (Drs. T.-U. W. Woo and F. M. Benes) reviewed all records and applied the criteria of Feighner *et al*. [32] for the diagnosis of schizophrenia and DSM III-R criteria for the diagnosis of schizoaffective disorder. Seventeen of the 20 schizophrenia subjects were on antipsychotic medications at the time of death. Some of these subjects were receiving concomitant psychotropic medications, such as anticonvulsants, mood stabilizers, antidepressants or anxiolytics (Additional file 1). None of the normal control subjects was on any psychotropics at the time of death. Toxicology data together with clinical information confirmed that none of the subjects in each group suffered from any substance-related disorders at the time of death.

### Double *In Situ *Hybridization

#### Tissue Preparation

Tissue blocks, each about 3 mm in thickness, were removed from Brodmann's Area 9 of fresh brain specimens and fixed in 0.1% paraformaldehyde in ice-cold 0.1 phosphate buffer saline (PBS; pH 7.4) for 90 minutes, immersed in 30% sucrose in the same buffer overnight, and then frozen in Tissue Tek OCT (Sakura Finetek, Torrance, CA). Sections of 10 μm were cut on a cryostat, mounted on slides, and stored at -70°C until use. Two sections per subject were used for *in situ *hybridization.

#### Riboprobe Preparation

##### Radiolabeled cRNA probe for NR2A

The complementary RNA (cRNA) probes were transcribed *in vitro *from full-length complementary DNA (cDNA) clones of the rat NR2A (Genbank Accession No. M91561) subunit (kindly provided by Dr. Christine Konradi), which is 89% identical to the human sequence, as described previously [19,20]. This same riboprobe was used in our previously published studies [19,20,33]. Briefly, the probe was derived from a cDNA spanning nucleotides 1185 to 2154 within the coding region of the gene. A corresponding sense probe was also generated and hybridization of the sense probe resulted in no specific labeling. Radiolabeled cRNA was prepared by first drying down [^35^S]UTP (500 mCi/ml of probe, Perkin Elmer Life and Analytical Sciences Inc, Boston, Mass) in a DNA Speed-Vac (Savant, Farmingdale, NY). 100 ng/ml of the cDNA template, 0.1 M dithiothreitol (DTT), 3 U/ml of RNasin, 5 mM NTPs, 0.8 U/ml T3 or T7 polymerases (for antisense and sense probe respectively), and 5× transcription buffer were then added. The transcription mixture was subsequently incubated at 37°C for 2 hours. The cDNA template was digested by incubating the mixture with R1Q DNAse at 37°C for 15 minutes. Unincorporated NTPs were removed by running the mixture through a Stratagene Nuc-Trap (La Jolla, CA) push column. The eluate was collected, and probe concentration was determined by scintillation counting. The probe was stored at -20°C until use.

##### Digoxigenin (DIG)-labeled cRNA probe for PV

A single PCR product of 510 bp (spanning nucleotides 51-560) within the coding region of the human parvalbumin gene (Genbank Accession No. NM_002854.2) was amplified from human brain cDNA. DIG-UTP-labeled cRNA probes were transcribed using 100 ng of linearized parvalbumin cDNA subclones in the presence of 0.1 M DTT, 3 U/ml RNasin, 0.8 U/ml of T3 and T7 RNA polymerases, 10 mM of ATP, CTP, and GTP, 6.5 mM of UTP, and 3.5 mM of DIG-labeled UTP (Roche, Indianapolis, IN). The mixture was incubated at 37°C for 2 hours. cDNA template was digested with RQ1 DNase. The sense probe was also generated as control.

#### Hybridization

Sections were hybridized in a buffer consisting of 50% formamide, 0.1% yeast transfer (t)RNA, 10% dextran sulfate, 1× Denhardt's solution, 0.5 M/l ethylenediamine tetracetic acid (EDTA), 0.02% sodium dodecyl sulfate (SDS), 4× saline-sodium citrate buffer (SSC), 10 mM dithiothreitol (DTT), and 0.1% single stranded DNA (ssDNA) at a final concentration of 0.4 ng probe/ml hybridization buffer. They were then post-fixed in 4% paraformaldehyde for 10 minutes and incubated in 0.1 M triethanolamine (TEA) for 5 minutes at room temperature before being dehydrated in a graded series of ethanol. Probes were then added to slides for hybridization in a prewarmed, humidified dish. Sections were covered with coverslips and incubated at 56°C for 12 hours in a humid chamber. At the end of hybridization, coverslips were soaked off in 4× SSC in the presence of 100 μl of β-mercaptoethanol (βMer). Tissue was then incubated in 0.5 M NaCl/0.05 M phosphate buffer for 10 minutes, 0.5 M NaCl with 0.025 mg/ml RNaseA at 37°C for 30 minutes, followed by a high stringency wash with a solution containing 50% formamide, 0.5 M NaCl/0.05 M phosphate buffer, and 100 ml βMer at 63°C for 30 minutes. Sections were finally washed overnight in 0.5× SSC with 20 mM βMer at room temperature.

#### Visualization of DIG labeling

After incubation in blocking buffer (100 mM Tris-HCl, 150 mM NaCl [pH 7.5]), the sections were placed in buffer containing 3% normal donkey serum + 0.3% Triton X100), and incubated overnight at 4°C in buffer containing 1:200 dilution of sheep anti-DIG antibody conjugated with peroxidase enzyme (Roche Diagnostics, Indianapolis, IN). Sections were washed in buffer and a peroxidase reaction product was localized 3,3'-diaminobenzidine tetrahydrochloride in the presence of H_2_O_2_.

#### Emulsion Autoradiography

Slides were apposed to X-ray film (Kodak Biomax MS) for 10 days to determine the presence of sufficient autoradiographic signal. The microscope slides were then dipped in emulsion, dried and exposed at 4°C in the dark for 5 weeks. They were then developed in Kodak D-19 developer, counterstained and coverslipped.

#### Quantification of Cell Density

The slides were coded so that the diagnosis of each case was unknown to the investigator (BKYB). [^35^S]-labeling of NR2A mRNA appeared as clusters of silver grains after emulsion autoradiography processing. Quantification was performed as previously described [19,20,33,34]. DIG labeling, in the form of a brown reaction product, was visualized under a bright field microscope equipped with polarizing filters to enhance the optical density of the reaction product. Neurons that were single labeled with DIG and those that were double labeled with DIG and [^35^S] were identified on images captured on a computer screen using a microscope (Laborlux, Leica Microsystems, Wetzlar, Germany) fitted with a solid CCD video camera and connected to a Bioquant Nova Image Analysis System (R&M Biometrics, Memphis, TN). A touch counting subroutine was used to determine the distribution of both single and double-labeled neurons within a 250 μm-wide cortical traverse extending from the pial surface to the white matter border by using a X100 oil immersion objective lens at a final magnification of 1,000×. Two cortical traverses per section and therefore four cortical traverses per case were analyzed. For each case, cell counts averaged from the four cortical traverses were used in statistical analysis, so that each individual had only one representative measurement at each cortical depth. Neighboring sections were stained with cresyl violet for determination of laminar boundaries. Densities of both single and double-labeled neurons for each cortical layer were then obtained by dividing cell counts by laminar areas. Prior to the actual data collection, intrarater reliability, as assessed by counting and recounting profiles in the same column, was established to be above 95%.

To quantify the expression level of mRNA for the NR2A subunit in individual PV cells, the area occupied by each grain cluster was carefully outlined using a cursor displayed on the computer monitor, as previously described [19,20,33,34]. The cluster area was measured by highlighting the grains with a thresholding subroutine. All parameters were held constant throughout the entire course of quantification. The area covered by autoradiographic grains within the cluster area was automatically computed by the Bioquant software based on the threshold value and was represented as a pixel count for NR2A transcript expression level. The pixel count was expressed as a function of cluster area after correcting for background (i.e. pixel count of the area covered by autoradiographic grains per unit area in square micrometers in the white matter). The average NR2A expression level in PV neurons was computed for each cortical layer.

### vGluT1 Immunohistochemistry

Tissue blocks containing Brodmann's Area 9 were fixed in ice cold 4% paraformaldehyde overnight, cryoprotected in 30% sucrose, embedded in Tissue Tek OCT (Sakura Finetek, Torrance, CA), and sectioned at 40 μm on a microtome. The entire experiment was completed in 2 runs. During each run, two sections per subject were used and all sections from each subject pair were processed together. Free-floating sections were rinsed for 15 min in 0.3% H_2_O_2_, 0.5% Triton-X, and 10% methanol in PBS. They were then pre-incubated for 30 minutes in 5% normal horse serum and 0.3% Triton-X in PBS. Sections were subsequently incubated at room temperature overnight in a polyclonal rabbit anti-vGluT1 antibody (1:1000) diluted in 2% normal horse serum and 0.5% Triton-X in PBS. The specificity of the antibody has been extensively characterized [30,31,35,36]. After incubation, sections were rinsed in PBS and incubated at room temperature for 2 hours in biotinylated donkey anti-rabbit IgG (1:500; Jackson ImmunoResearch Laboratories, West Grove, PA) in 2% normal horse serum and 0.5% Triton X-100 in PBS. They were then rinsed in PBS and incubated for 2 hours in ABC Elite (1:500; Vector Laboratories, Burlingame, CA) in PBS. vGluT1 elements were visualized with 0.4 mg/mL DAB (Sigma, St. Louis, MO), 0.0006% hydrogen peroxide, and 0.4 mg/mL nickel ammonium sulfate dissolved in PBS. Sections were mounted, air-dried, dehydrated, cleared in xylenes and coverslipped.

#### Quantification

All microscopic analyses were conducted under strictly blind condition. Sampling was performed in layers 3 and 5 within two 500 μm-wide traverses, 500 μm apart from one another. Laminar boundaries were determined by comparing with neighboring Nissl-stained sections. Quantification was performed using a Leica Laborlux microscope equipped with a solid-state video camera and Bioquant Nova Image Analysis System. Using a X100 oil immersion objective lens, the areas covered by vGluT1-immunoreactive boutons were outlined, computed and represented as pixel counts. Density measure per section was computed by averaging the measurements obtained from the two 500 μm-wide traverses. The density for each subject was obtained by averaging the measurements from all 4 sections.

### Statistical Analysis

The densities of PV mRNA+ and PV mRNA+/NR2A mRNA+ neurons and the NR2A mRNA expression level in PV+ neurons were compared between both groups across layers 2 through 6 using repeated-measures analysis of variance (ANOVA) with diagnosis and layer as main effects. Layer 1 was not included as no PV+ neurons were found in this layer. The density of vGluT1-immunoreactive boutons was compared between the two diagnosis groups, using unpaired t-tests. Analyses were performed using the JMP 5.1 (SAS Institute, Cary, NC) software program and all statistical tests were conducted with α = 0.05.

#### Confounding Variables

For both *in situ *hybridization and immunohistochemical experiments, we evaluated the effects of confounding variables, such as age, PMI, brain, pH, freezer storage time and exposure to antipsychotic medication (expressed as chloropromazine equivalent dose or CED) using analysis of covariance (ANCOVA). Because none of the conclusions derived from our findings were affected by the ANCOVA analysis, only results from repeated-measures ANOVAs are reported. In addition, Pearson's correlation was used to assess if there was any linear relationship between cell, grain or vGluT1-immunoreactive bouton densities and any of the continuous variables. Effects of hemispheric laterality and sex were evaluated by using 2-tailed unpaired t tests to compare the measures from the two hemispheres within individual groups.

## Results

### Distribution of PV mRNA+ and PV mRNA+/NR2A mRNA+ Neurons

**Figure 1:**
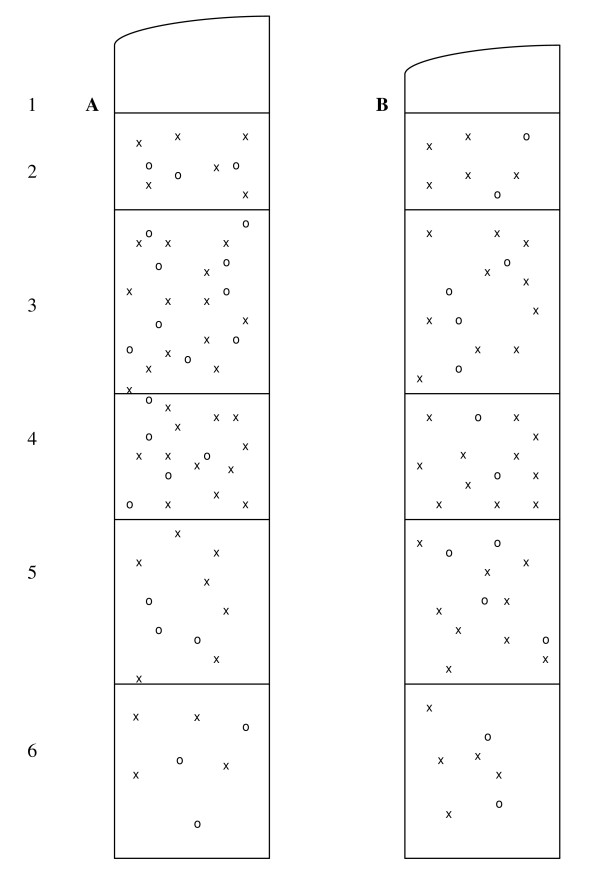
**Plots of PV+/NR2A+ neurons from representative sections from a normal control (A) and a schizophrenia subject (B). Numbers depict cortical layers**. X = PV mRNA-expressing neurons. O = PV neurons that co-express NR2A mRNA

Neurons that expressed PV mRNA were present predominantly in layers 3, 4 and 5 and were also observed in layers 2 and 6, but were absent from layer 1. The PV mRNA+/NR2A mRNA+ neurons seemed to be most concentrated in layers 3 and 4 (Figure 1).

### Density of PV mRNA+/NR2A mRNA+ Neurons

**Figure 2:**
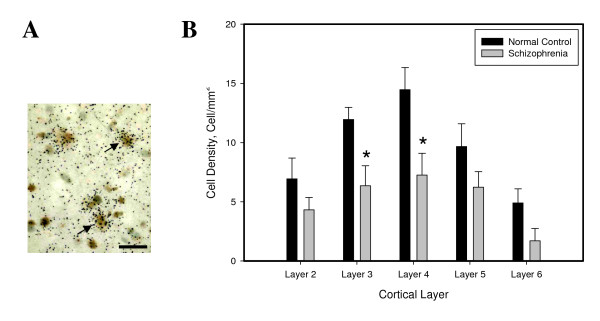
**(A) Photomicrograph showing examples of PV+/NR2A+ neurons**. Scale bar = 20 μm. (B) Mean (± SEM) density of PV+/NR2A+ neurons is significantly decreased in layers 3 and 4 in the PFC in schizophrenia.

The effect of diagnosis was significant (*F *= 6.62; df = 1, 38; *P *= 0.01). Furthermore, this effect was layer specific (*F *= 3.75; df = 1, 38; *P *= 0.006). Thus, in the subjects with schizophrenia, the density of the double-labeled neurons was significantly decreased by 48% and 50% in layers 3 (*t *= -2.11, *P *= 0.04) and 4 (*t *= -2.15, *P *= 0.03), respectively (Figure 2).

### Density of PV mRNA+ Neurons

Consistent with previous observations [26,37], we found that the number of cells that expressed a detectable level of PV mRNA was not altered in subjects with schizophrenia (*F *= 3.35; df = 1, 38; *P *= 0.07). The use of digoxigenin to label PV mRNA, however, precluded us from addressing any possible alteration in transcript expression level per neuron, as has been reported previously [26].

### Cellular Expression of NR2A mRNA

**Figure 3:**
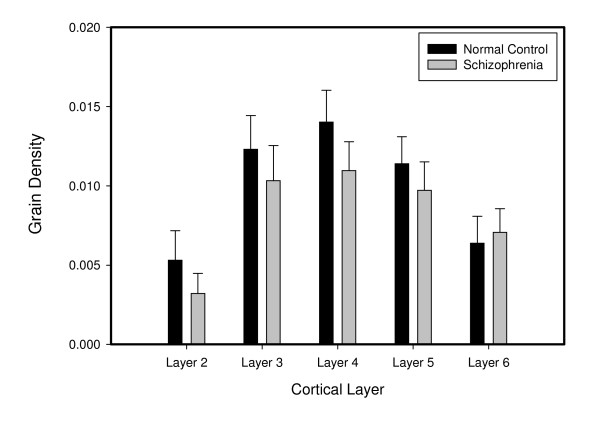
**Mean (± SEM) density of silver grains over PV+ neurons in the PFC is not different between the two subject groups, suggesting that the expression level of NR2A mRNA in PV cells is unchanged in schizophrenia**.

The density of silver grains per neuron did not differ between the schizophrenia and normal control groups (Figure 3). Furthermore, the distributions of the frequency histograms of NR2A mRNA expression level per PV cell did not differ between the two study groups (data not shown). Taken together, it can be concluded that in the PV cells that expressed a detectable level of NR2A mRNA, the amount of transcript expression was unaltered in schizophrenia.

### vGluT1 Immunoreactive Boutons

**Figure 4:**
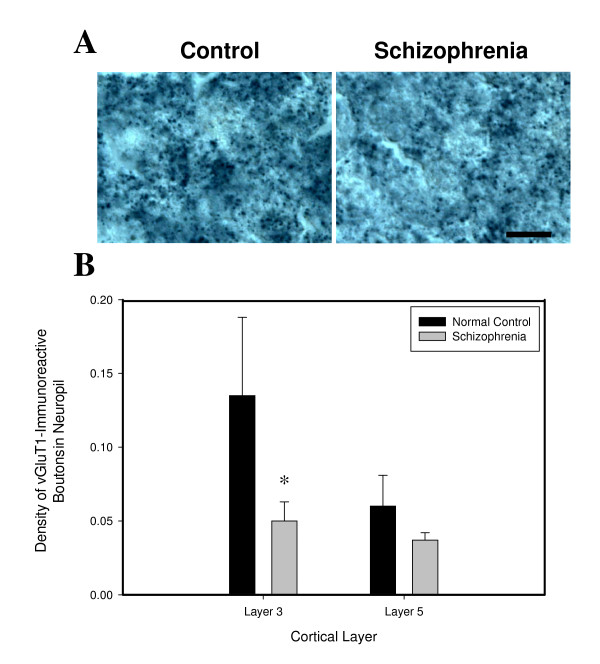
**(A) Photomicrographs showing vGluT1-immunoreactive boutons in layer 3 of the PFC**. Scale bar = 100 μm. (B) Density of vGluT1-immunoreactive boutons is significantly decreased in layer 3 of the PFC in schizophrenia.

The density of vGluT1-immunoreactive boutons in layer 3 of the PFC was significantly decreased by 79% in subjects with schizophrenia (Figure 4; *t *= 2.07, *P *= 0.05).

### Confounding Variables

Potential confounding variables (i.e. age, PMI, brain pH, freezer storage time, hemispheric laterality, antipsychotic drug exposure, sex and laterality) were evaluated with respect to the densities of PV mRNA+ and PV mRNA+/NR2A mRNA+ neurons, and vGluT1-immunoreactive boutons. None of these factors appear to have influenced our results.

## Discussion

We have extended our previous finding of reduced NR2A mRNA expression in GABA neurons in schizophrenia [19,20] to demonstrate that, in the PFC, this reduction occurs in a subset of PV-containing neurons. Furthermore, the density of axonal boutons that were immunoreactive for vGluT1, which is localized to presynaptic terminals furnished by cortically-originated glutamatergic projections [30,31,38], also appears to be decreased. Together these observations suggest that innervation of PV neurons by corticocortical glutamatergic projections via NMDA receptors may be deficient in schizophrenia.

The observation of decreased density of the NR2A-expressing PV neurons might represent a loss of these neurons, but previous studies suggest that cell loss does not seem to be a prominent feature in the PFC in schizophrenia, at least not in large scale [39,40]. Furthermore, consistent with previous observations [26,37], in this study, we found that the density of PV neurons was unaltered in schizophrenia subjects. Taken together, our finding is most consistent with the interpretation that, in a subset of PV neurons, the expression of NR2A is reduced to a level that is no longer experimentally detectable.

It has long been known that treatment with NMDA receptor antagonists produces a syndrome that is highly reminiscent of the clinical picture of schizophrenia [41,42], and these data led to the NMDA receptor hypofunction model [43]. The paradoxical excitotoxic effects originally observed by Olney and Farber with NMDA antagonists were explained, at least in part, by blockade of the NMDA receptors that are located on GABA neurons, which have been shown to be some 10-fold more sensitive to NMDA receptor antagonists than the NMDA receptor on pyramidal neurons [43-45]. To this end, a recent study by Kinney and colleagues found that the amount of NR2A mRNA expressed in cultured PV neurons was 5-fold higher than in pyramidal cells [27]. Similarly, compared to other NMDA subunits, the expression of NR2A appears to be prevalent in PV neurons [29]. Moreover, NR2A, but not NR2B selective antagonists, down-regulate GAD_67 _and PV mRNA expression in cultured PV neurons [27]. Finally, NMDA antagonism has been found to decrease the cellular expression of PV [46-48] and the number of axo-axonic cartridges [49]. Taken together, our finding of reduced NR2A expression in PV neurons may, at least in part, contribute to the reduction in the expression of GAD_67 _and PV and the decrease in the density of axon cartridges in schizophrenia [26,50-52].

The expression of the mRNA for vGluT1 has been found to be decreased in the PFC in schizophrenia [53], but negative results have also been reported [54]. At the protein level, using Western blot analysis, vGluT1 has been shown to be unaltered in the PFC, but decreased in the anterior cingulate cortex in schizophrenia [54]. In this study, we found that the density of the axonal boutons that were immunoreacvtive for vGluT1 was decreased in layer 3 of the PFC in subjects with schizophrenia. A likely explanation for the seeming discrepancy between this study and the previous report [54] may be attributable to methodological differences, i.e. immunohistochemistry in combination with quantification of axonal boutons afforded us a higher sensitivity in detecting differences in the present study. In the context of our finding, it is interesting to note that, a single polymorphism in the neuregulin-1 gene, a schizophrenia risk gene [55], has recently been reported to be associated with vGluT1 expression in human postmortem brains, with the at-risk allele predicting decreased expression of the glutamatergic marker [56].

Our observation of reduced vGluT1-immunoreactive boutons in layer 3 indicates that the number of glutamatergic axon terminals may be decreased in schizophrenia. An alternative interpretation of this finding is that the boutons may remain structurally intact (i.e. their number does not change), but they are functionally altered (i.e. vGluT1 expression is reduced). Because dendritic spines are the most common target of glutamatergic terminals [57], and because density of spines on layer 3 pyramidal cells has been found to be decreased by ~20% in schizophrenia [58], it would appear that our finding of reduced vGluT1 immunoreactivity reflects, at least in part, a loss of axon terminals. However, the magnitude of spine loss of 20% is insufficient to account for the current observation of a 79% reduction in vGluT1 immunoreactivity. Because up to 85% of all asymmetric synapses (i.e. synapses formed by glutamatergic terminals) in the cortex are formed almost exclusively on dendritic spines [59], and this amount is similar to the magnitude of reduction in vGluT1-immunoreactive profiles observed in this study, one interpretation of our finding is that the majority of the glutamatergic terminals that target pyramidal neurons in layer 3 of the PFC are functionally disturbed in schizophrenia and, among these terminals, approximately 20% are lost. Alternatively, but not mutually exclusively, glutamatergic terminals that target neural structures other than spines may also be affected. Interestingly, aside from dendritic spines, the PV-containing neurons constitute the prime target of these terminals [11]. Given our finding of reduced NR2A expression in these neurons, it seems likely that glutamatergic terminals that are presynaptic to PV neurons may also be disturbed. In order to definitively address this hypothesis, however, double immunolabeling in combination with confocal or electron microscopy will be required.

Deficient glutamatergic inputs to PV neurons could, via disinhibition [60], lead to increased activity of excitatory circuits that are postsynaptic to these neurons, rendering them more vulnerable to excitotoxic or oxidative injury [8,43,61,62]. Short of leading to cell death, available evidence suggests that cellular injury can be manifested in the form of neuritic and synaptic atrophy [63-68] and hence may contribute to the observed reduction in dendritic spines [58,69], pyramidal cell somal area [70], synaptic markers [71-76] and volume of neuropil [39] in schizophrenia. In addition, PV neurons are known to play a critical role in the orchestration of oscillation of pyramidal cell circuits in the gamma frequency band (30-100 Hz), which is thought to be a mechanism that supports information integration [77-80]. Recent studies increasingly converge upon the notion that gamma oscillation disturbances represent a prominent pathophysiologic feature of schizophrenia [81-83]. In addition, animal studies have shown that NMDA receptor blockade robustly disrupts gamma rhythms in the entorhinal cortex [84] and it is speculated that this disruption may be mediated by the NMDA receptors on the PV-containing neurons [84]. Taken together, reduced glutamatergic inputs onto PV neurons via NMDA receptors may in part contribute to aberrant gamma oscillations in schizophrenia. However, it is important to note that the contribution of NMDA receptor to excitatory neurotransmission onto PV neurons varies across brain regions [85,86]. This may explain why the effects of NMDA antagonism on gamma oscillations are highly region-dependent [87].

## Conclusion

Convergent lines of evidence suggest that glutamatergic neurotransmission on PV-containing neurons via NMDA receptors appears to be deficient in schizophrenia. Altered NMDA receptor expression is not restricted to PV neurons. For example, the expression of NR2A mRNA has also been found to be altered in the inhibitory neurons that contain another calcium buffer calbindin [33], which target the dendrites of pyramidal neurons. Likewise, altered glutamate receptor expression in inhibitory neurons is not confined to the NMDA class; the expression of the mRNA for the GluR5 kainate receptor in GABA neurons, for instance, has also been found to be altered [88]. It seems clear that more studies are needed before we can better define how cortical circuits are disturbed in schizophrenia, and the potential functional consequences of these disturbances. Such knowledge will provide a neurobiologic framework within which it may be possible to conceptualize rational therapeutic strategies that aim at normalizing or recalibrating the malfunctioned brain circuits [89,90].

## Competing interests

The authors declare that they have no competing interests.

## Authors' contributions

BKYB and MPL conducted the experiments and the microscopic quantification. JFK assisted in data collection. BKYB performed data analysis. BKYB, TK and TUWW reviewed and discussed the findings. BKYB and TUWW wrote the manuscript. All authors read and approved the final manuscript.

## Pre-publication history

The pre-publication history for this paper can be accessed here:

http://www.biomedcentral.com/1471-244X/9/71/prepub

## Supplementary Material

Additional file 1 Supplementary table S1 Click here for file
