# Supplementary material for: Glutamatergic deficits and parvalbumin-containing inhibitory neurons in the prefrontal cortex in schizophrenia
Source: BMC Psychiatry. 2009 Nov 16;9:71. doi: 10.1186/1471-244X-9-71 (PMC2784456; doi:10.1186/1471-244X-9-71)
Supplement: Additional file 1 — Supplementary table S1 [file 1471-244X-9-71-S1.DOC]

| ***Normal Control*** | | | | | | | | | |
| --- | --- | --- | --- | --- | --- | --- | --- | --- | --- |
| Case | Age | Sex | Race | Side | pH | PMI | CED | Cause of death | Psychotropics  Received at time of death |
| 1 | 49 | M | W | L | 6.76 | 24.6 |  | Myocardial infarction | None |
| 2 | 37 | M | W | R | 6.68 | 18.8 |  | Electrocution | None |
| 3 | 54 | M | W | L | 6.53 | 24.2 |  | Cardiopulmonary arrest | None |
| 4 | 78 | F | W | R | 6.22 | 14.1 |  | Myocardial infarction | None |
| 5 | 53 | M | W | R | U | 20.2 |  | Cardiopulmonary arrest | None |
| 6 | 65 | F | W | R | 6.4 | 24.3 |  | Lung cancer | None |
| 7 | 89 | M | W | R | 6.39 | 7.42 |  | Cancer | None |
| 8 | 69 | M | W | R | 6.88 | 15.3 |  | Respiratory failure | None |
| 9 | 74 | F | W | L | U | 12.5 |  | U | None |
| 10 | 66 | F | W | R | 6.03 | 7.4 |  | Cancer | None |
| 11 | 42 | M | W | L | 6.78 | 18.3 |  | Myocardial infarction | None |
| 12 | 78 | F | W | R | 6.67 | 23.9 |  | Breast cancer | None |
| 13 | 40 | M | W | L | 6.24 | 16.6 |  | Myocardial infarction | None |
| 14 | 67 | M | W | L | 6.42 | 22.3 |  | Cardiopulmonary arrest | None |
| 15 | 70 | F | W | L | 6.26 | 22.5 |  | Liver cancer | None |
| 16 | 66 | M | W | R | 6.76 | 18.7 |  | Myocardial infarction | None |
| 17 | 79 | M | W | L | 6.74 | 20.9 |  | Cancer | None |
| 18 | 38 | M | W | L | 6.53 | 28.8 |  | Myocardial infarction | None |
| 19 | 70 | F | W | R | 6.59 | 15 |  | Cardiac arrest | None |
| 20 | 29 | M | W | L | U | 19 |  | U | None |
| Mean(±SD) | 60.4 ±17.3 | 13M:7F |  | 10L:10R | 6.58 ±0.25 | 18.7 ±5.5 |  |  |  |

Abbreviations are as follows: PMI = post-mortem interval, CED = chloropromazine equivalent dose, U = unknown or unavailable, M = male, F = female, L = left, R = right, W = white.

| ***Schizophrenia*** | | | | | | | | | |
| --- | --- | --- | --- | --- | --- | --- | --- | --- | --- |
| Case | Age | Sex | Race | Side | pH | PMI | CED | Cause of death | Psychotropics  Received at time of death |
| 21 | 85 | F | W | R | U | 15.7 | 150 | Sepsis | Risperidone, lorazepam |
| 22 | 48 | F | W | L | 6.63 | 33.8 | 450 | Cardiac arrest | Risperidone, divalproex |
| 23 | 44 | M | W | L | 6.2 | 19 | 266 | Pneumonia | Clozapine |
| 24 | 89 | F | W | L | U | 13.5 | 20 | Pneumonia | Trifluoperazine |
| 25 | 78 | F | W | L | 6.81 | 13.4 | 750 | Sinus node disease | Haloperidol, lithium, cogentin |
| 26 | 61 | M | W | R | 6.68 | 19.9 | 300 | Sepsis | Clozapine |
| 27 | 61 | F | W | R | 6.14 | 11 | 150 | Myocardial infarction | Paroxetine, clonazepam, clozapine |
| 28 | 84 | F | W | R | 6.14 | 25.8 | U | Cardiac arrest | None |
| 29 | 26 | M | W | R | 6.75 | 16 | 357 | Suicide by hanging | Fluphenazine, decanoate |
| 30 | 55 | F | W | R | 6.52 | 18 | U | Lung cancer | None |
| 31 | 47 | M | W | R | 6.57 | 19.2 | U | Lung cancer | Clonazepam, hydroxyzine |
| 32 | 73 | F | W | R | 6.08 | 24 | 600 | Lung cancer | Risperidone, fluoxetine, clorazepate, midazolam |
| 33 | 49 | M | W | L | 6.6 | 19 | 500 | Suicide by hanging | Haloperidol decanoate, lorazepam |
| 34 | 63 | M | W | R | 6.55 | 22.3 | 500 | Cardiac arrest | Cloazapine, haloperidol, lorazepam, trazodone |
| 35 | 72 | F | W | R | 6.65 | 21.7 | 400 | Ovarian cancer | Risperidone, paroxetine |
| 36 | 66 | M | W | R | 6.43 | 22.1 | 1000 | Emphysema | Haloperidol |
| 37 | 83 | F | W | R | 6.91 | 23.2 | 2000 | Gastrointestinal bleed | Haloperidol decanoate |
| 38 | 46 | F | W | L | 6.31 | 18.5 | 200 | Sepsis | Olanzapine, divalproex |
| 39 | 42 | M | W | R | 6.64 | 27.1 | U | Leukemia | None |
| 40 | 31 | M | W | R | 6.46 | 14 | 600 | U | Risperidone, olazapine, buproprion |
| Mean(±SD) | 60.2 ±16.7 | 9M:11F |  | 6L:14R | 6.65 ±0.28 | 19.8 ±5.4 | 433±468 |  |  |

Abbreviations are as follows: PMI = post-mortem interval, CED = chloropromazine equivalent dose, U = unknown or unavailable, M = male, F = female, L = left, R = right, W = white.
